# Formulation and Optimization of Monolithic Fixed-Dose Combination of Metformin HCl and Glibenclamide Orodispersible Tablets

**DOI:** 10.1155/2020/3546597

**Published:** 2020-02-18

**Authors:** Anteneh Belayneh, Fantahun Molla, Getu Kahsay

**Affiliations:** ^1^Department of Pharmacy, College of Health Sciences, Debre Markos University, Debre Markos, Ethiopia; ^2^Department of Pharmaceutics, School of Pharmacy, College of Health Sciences, Mekelle University, Mekelle, Ethiopia; ^3^Department of Pharmaceutical Analysis and Quality Assurance, School of Pharmacy, College of Health Sciences, Mekelle University, Mekelle, Ethiopia

## Abstract

The treatment of type II DM involves the use of combination of drugs, especially at the chronic stage. However, the pill burden of this combination therapy combined with swallowing difficulties, occurring at a later stage of DM, has been the major challenge for successful treatment outcomes. This study was aimed at formulating and optimizing a monolithic fixed-dose combination (FDC) of metformin (MET) and glibenclamide (GLB) orodispersible tablets (ODTs) to overcome both the pill burden and swallowing problems. The FDC ODTs were prepared by the melt granulation technique using polyethylene glycol (PEG) 6000 as a binding agent and crospovidone as a superdisintegrant. In the preliminary study, the effects of sodium lauryl sulphate (SLS), PEG 6000, crospovidone, and compression force on friability, disintegration time, and drug release of tablets were investigated. The FT-IR studies showed that there were no incompatibilities between MET and GLB as well as within excipients. The preliminary studies revealed that PEG 6000 and compression force significantly affect both the friability and the disintegration time, while SLS and crospovidone only affect the disintegration time. Therefore, the effects of PEG 6000, crospovidone, and compression force were further studied and optimized using the central composite design. Accordingly, the most desirable optimal values were obtained at 3.82% of PEG 6000, 9.83% of crospovidone, and 10.6 kN compression force having a friability of 0.302% and a disintegration time of 18.7 seconds. From these results, it can be concluded that a monolithic FDC of MET and GLB ODTs having adequate mechanical strength and faster disintegration time was successfully formulated.

## 1. Introduction

Diabetes mellitus (DM) is one of the most prevalent noninfectious diseases characterized by a collection of metabolic disorders which causes hyperglycemia. It is associated with abnormalities in protein, carbohydrate, and fat metabolism which result in severe complications such as microvascular, macrovascular, and neuropathic problems [1]. There were more than 422 million people who had DM in 2014, accounting for 8.5% of the adult population. In the same year, 3.8 million deaths occurred due to DM [2]. From all these deaths, 90–95% were due to type II DM [3].

Health care providers usually prescribe metformin and glibenclamide together as a first-line therapy during the management of type II DM patients. This combination is also used when a monotherapy of each drug failed to effectively manage the case [4, 5]. However, the use of two or more separate tablets for the management of DM has exposed patients to pill burden which often leads to poor treatment adherence. The WHO stated that more than 50% of type II DM treatment failure was due to poor adherence to medications. The major causes of this poor adherence, around 63%, were polypharmacy and pill burden [5]. In addition to pill burden, the swallowing problem has been the major challenge for the DM patients. For instance, according to the study by Saleh and Sulieman [6], about 44% of type II DM patients suffered from the swallowing problem of conventional tablets and hard gelatin capsules. To improve the treatment adherence of DM patients, a strategy aiming at reducing pill burden and overcoming swallowing difficulty is required. One potential strategy is formulation of fixed-dose combination (FDC) orodispersible tablets (ODTs) to solve both pill burden and swallowing problem. Fixed-dose combinations (FDCs) are a combination of two or more APIs in a single dosage form, which is manufactured in fixed doses, and ODTs are tablets that disintegrate and dissolve rapidly in the saliva, with a few seconds or minutes without the need of water or chewing [7, 8]. Thus, the aim of this study was to develop the monolithic FDC of metformin hydrochloride and glibenclamide ODTs.

## 2. Materials and Methods

### 2.1. Materials

Metformin hydrochloride (Auro Laboratory Pvt. Ltd., India), polyethylene glycol (PEG) 6000 (Nanjing Well Pharmaceuticals, China), crospovidone (Ludwigshafen Chemicals, China), microcrystalline cellulose, Avisol 102 (Ankit Pulps and Boards Pvt. Ltd., India), aspartame (Sinolight Chemicals Co., Ltd., China), magnesium stearate (Anhui Menovo Pharmaceutical Co., Ltd., China), colloidal silicon dioxide (Boai NKY Pharmaceutical Co., Ltd., China), and sodium lauryl sulphate (LRC Speciality Chemicals, India) were obtained as a gift from Addis Pharmaceutical Factory (APF), Adigrat, Ethiopia. Glibenclamide micronized powder (Cadila Pharmaceuticals Ltd., Ankleshwar, India) was kindly donated by Cadila Pharmaceutical Manufacturing PLC, Addis Ababa, Ethiopia. Potassium dihydrogen orthophosphate (Loba Chemie pvt ltd, India), disodium hydrogen orthophosphate (Titan Biotech Ltd., India), ammonium acetate (Choan Chemicals, China), amaranth red (Panjit, India), methanol (Dasit Group, France), and distilled water were purchased from a local market. Methanol and water were of HPLC grade, and all other chemicals used in this study were of analytical grade.

### 2.2. Methods

#### 2.2.1. Drug-Drug and Drug-Excipient Compatibility Study

Drug-drug, MET : GLB (100 : 1), and drug-excipient, MET : GLB : PEG : SLS (100 : 1 : 10 : 1.4), interaction studies were carried out using Fourier transform infrared spectroscopy (FT-IR) (SHIMADZU FT-IR-8400S, Japan). The FT-IR spectra were conducted for pure MET HCl, pure GLB, mixture of MET HCl and GLB, pure PEG 6000, pure SLS, and mixture of two drugs with PEG 6000 and SLS. The IR spectrum was collected with 20 scans at a resolution of 4 cm^−1^ at 25°C. Scanning was performed between wavenumbers 4000 and 400 cm^−1^.

#### 2.2.2. Preparation of Fixed-Dose Combination Orodispersible Tablets

The melt granulation method as described by Bareth et al. [9] with slight modifications was used to prepare the ODTs. Accordingly, PEG 6000 was weighed, added in a porcelain dish, and melted at 65°C in a water bath (HH-S4, Germany) until homogenized. To the melted mass, the accurate quantity of geometrically mixed metformin HCl and glibenclamide powder was mixed with continuous stirring. Then, the previously mixed mixture of crospovidone (half amount), microcrystalline cellulose (MCC), aspartame, and SLS was added to the molten drug-PEG 6000 mixture and stirred well to mix. The mixture was then allowed to solidify at 25°C by spreading in a thin layer, pulverized in a mortar, and sieved through a 45-mesh screen (Fritsch, Germany) to form granules. The remaining amount of crospovidone was mixed with the prepared granules. Finally, magnesium stearate and colloidal silicon dioxide were mixed to the prepared granules and compressed by a single punch tablet machine (Riva, Germany) at different compression force to the average weight of 350 mg with tablet composition, as shown in Table 1.

### 2.3. Evaluation of Precompression Parameters

#### 2.3.1. Density and Flow Properties

Bulk and tapped densities were measured by the conventional tapping method using a 200 mL graduated measuring cylinder as per the following equations:

(1) $$\begin{array}{c} D_{b}=\frac{W}{V_{b}}, \end{array}$$

(2) $$\begin{array}{c} D_{t}=\frac{W}{V_{t}}, \end{array}$$

where *D*_b_ is the bulk density (g/mL), *W* is the mass of granules (g), *V*_b_ is the initial volume of the granules (mL), *D*_t_ = tapped density in g/mL, and *V*_t_ = tapped volume of the granules in mL.

Hausner's ratio and compressibility index were calculated from tapped and bulk densities using the following equations:

(3) Hausner's ratio=DtDb,

(4) Carr's index=Dt−DbDt×100.

The angle of repose of the granule was done by allowing 30 gm of granules to flow through a machine funnel freely onto the surface and calculated according to the following equation:

(5) $$\begin{array}{c} \theta \;=\;\tan^{-1}\left(\frac{H}{R}\right), \end{array}$$

where *H* = height of the pile and *R* = radius of the pile.

#### 2.3.2. Evaluation of Postcompression Parameters

The prepared tablets were characterized with respect to the standard pharmacopoeial parameters.

#### 2.3.3. Tablet Thickness and Diameter

Twenty tablets from each formulation were randomly taken and measured for thickness and diameter using a thickness and diameter tester (ERWEKA, Germany).

#### 2.3.4. Weight Variation

Twenty tablets were randomly selected from each batch and individually assessed for their weight (WI) using an analytical balance (OHAUS AR3130, China). The mean weight (WA) was calculated. Finally, the percent weight variation was calculated using the following equation:

(6) $$\begin{array}{c} \%\text{ of weight variation }=\frac{\text{WA}-\text{WI}}{\text{WA}}\times 100\;. \end{array}$$

#### 2.3.5. Tablet Hardness

The hardness of ten tablets from each batch was determined using a hardness tester (YD-2 Tianjin Gouming Medicinal Equipment Co.,LTD , China).

#### 2.3.6. Friability Test

From each formulation, twenty tablets were accurately weighed, placed in the friability test chamber (FAB-2A Logan Instruments Corporation, Germany), and rotated at a speed of 25 rpm for 4 minutes. The tablets were dedusted and weighed again, and the percentage weight loss was then calculated using the following equation:

(7) $$\begin{array}{c} F=\frac{W0-W1}{W0}\times 100, \end{array}$$

where *F* = percentage friability, *W*0 = initial mass before the test, and *W*1 = final weight after the test.

#### 2.3.7. Wetting Time

Tablet wetting time was found as per the method described by Naik and Chandrasekhar [10]. Circular tissue papers were placed in a Petri dish containing 10 mL of amaranth red-coloured water. Then, six tablets were placed on the surface of the tissue paper. The time required for water to reach the upper surface of the tablet was noted as the wetting time.

#### 2.3.8. In Vitro Disintegration Time

An *in vitro* disintegration test was carried out using a USP disintegration apparatus (PT-Z5, Pharma Test, Germany) by taking six tablets from each batch. Each tablet was placed in a disintegration apparatus containing 900 mL of distilled water and maintained at 37 ± 2°C. The time taken for complete disintegration of the tablet with no particulate matter remaining in the mesh was recorded as the disintegration time [11].

#### 2.3.9. In Vitro Dissolution Studies

An *in vitro* dissolution study of tablets was conducted in a USP type II dissolution test apparatus (PTW5820D, Pharma Test, Germany) at a rotation speed of 100 rpm. Six tablets from each batch were randomly taken for the dissolution study. Each tablet was placed in a beaker containing 900 mL of phosphate buffer with pH 6.8. The temperature was maintained at 37 ± 0.5 C. A sample of 5 mL was withdrawn at 30 minutes for the single-point dissolution test for the preliminary tablets, and similar amounts of samples were withdrawn at 5, 10, 20, 30, and 45 minutes to study the release profile of the optimized tablets. Each sample withdrawn was replaced with an equal volume of the fresh dissolution medium at 37°C to maintain the sink condition. The samples withdrawn were filtered through a 0.45 *μ*m membrane filter and analyzed for the percentage of drug release using HPLC (Agilent, Germany). The percentage of drugs dissolved was calculated by comparing the peak areas obtained from the sample solution with the peak areas obtained from the standard solution using the following equation [12]:

(8) $$\begin{array}{c} \text{\% DR}=\frac{\text{Pu}\times \text{Cs }\left(\text{mg}/\text{mL}\right)\times 900\text{ mL}\times 100}{\text{Ps}\times \text{Lc }\left(\text{mg}\right)}, \end{array}$$

where %DR = percentage of drug release, Pu = peak area of the sample, Ps = peak area of the standard, Cs = concentration of the standard, and Lc = label claim of the drug.

#### 2.3.10. Drug Content Determination

For the estimation of the drug content (%), twenty tablets were selected randomly and the average weight was calculated. The tablets were crushed in a mortar, and an accurate weight equivalent to 250 mg of MET HCl and 2.5 mg of GLB was weighed and dissolved in adequate quantity of methanol to get 250 *μ*g/mL of metformin HCl and 2.5 *μ*g/mL of glibenclamide. The prepared solution was then filtered through a 0.45 *μ*m membrane filter, and the filtrate was analyzed using HPLC as per the following equation:

(9) $$\begin{array}{c} \text{quantity }\left(\text{mg}\right)=\frac{\mathrm{CVD}\left(\text{rU}/\text{rS}\right)}{N}, \end{array}$$

in which *C* is the concentration of the standard (mg/mL), *V* is the volume in mL used to prepare the assay preparation, *D* is the dilution factor, rU is the peak area of assay preparation, rS is the peak area of the standard, and *N* is the number of tablets used to prepare the assay preparation.

#### 2.3.11. HPLC Condition

The amount of MET HCl and GLB dissolved was determined using the validated HPLC method [13]. All chromatographic processes were carried out at 25°C, and the two drugs were separated using the isocratic elution system. The percentage of each active component dissolved was calculated by comparing the peak areas of the test solution with the peak areas of the standard solution. The following chromatographic conditions were applied.

The mobile phase used was a mixture of 0.1 M ammonium acetate solution and methanol in a ratio of 23 : 77 v/v. The standard stock solutions of MET HCl and GLB containing 1.0 mg/mL of each were prepared with methanol. From this stock solution, 150 *μ*g/mL standard solutions were prepared by further diluting with methanol for both MET HCl and GLB. Separation was achieved on a reversed-phase C_8_ column (250 × 4.6 mm, 5 *μ*m particle size) with a flow rate of 1.2 mL/min and injection volume of 10 *μ*L. The detector was adjusted to 230 nm.

#### 2.3.12. Calibration Curve and System Suitability Test

**Figure 1:**
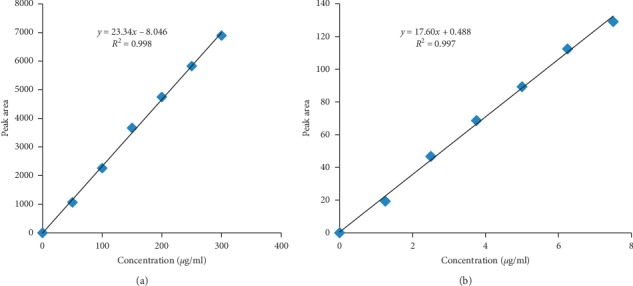
HPLC calibration curves for metformin HCl (a) and glibenclamide (b) using reference standards of MET and GLB in the phosphate buffer with pH 6.8.

Calibration curves were established by preparing series of dilutions from the standard stock solution to get a concentration range of metformin hydrochloride from 50 to 300 *μ*g/mL and glibenclamide from 1.25 to 7.50 *μ*g/mL. The above series solutions were injected into the HPLC system. The standard calibration curves for metformin hydrochloride and glibenclamide were constructed by plotting responses (peak areas of the drug) against their respective concentrations, as shown in Figure 1.

The system suitability test was performed to confirm that the HPLC system to be used was suitable for intended application. A standard solution containing 150 *μ*g mL^−1^ of MET and 150 *μ*g mL^−1^ of GLB was injected five times. The parameter retention time, % RSD, theoretical plates, and tailing factor were determined, as shown in Table 2.Data are expressed as mean + SD (*n* = 5).

## 3. Experimental Design

In the preliminary study, two-level full-factorial experimental design was applied to study the effect of three independent variables (PEG 6000, crospovidone, and compression force) on the response variables (disintegration time, friability, and percentage of drug release at 30 minutes). The factors were assessed at two levels (minimum and maximum), as shown in Table 3. According to this design, the number of formulations was 2^*k*^, where 2 indicates the level and *k* is the number of factors. For *k* = 3, 2^3^ = 8.

After the preliminary study, a central composite design (CCD) from the response surface methodology (RSM) was employed to optimize significant factors with respect to the response variables. The CCD with five coded levels, as shown in Table 4, was used to describe the nature of the response surface in the optimum region. According to this design, the total number of formulations was 2^*k*^ + 2*k* + *n*_o_, where *k* is the number of independent variables and *n*_o_ is the number of repetitions of experiments at the center point. For three factors, a total of 19 formulations (2^3^ + (2×3) + 5) were prepared in one block randomly to minimize bias due to uncontrollable factors.

### 3.1. Statistical Analysis

The statistical analysis was conducted using Origin 8 software (OriginLab Corporation, MA, USA). One-way analysis of variance (ANOVA) was applied for comparison of all results. Design-Expert 8.0.7.1 software (Stat-Ease, Corp., Australia) was employed to demonstrate graphically the influence of each factor on responses and to indicate the optimum level of factors. Each test was done in triplicate, and results were presented as the mean and standard deviation. A statistically significant difference was considered when *P* < 0.05.

## 4. Results and Discussion

### 4.1. Drug-Drug and Drug-Excipient Compatibility

**Figure 2:**
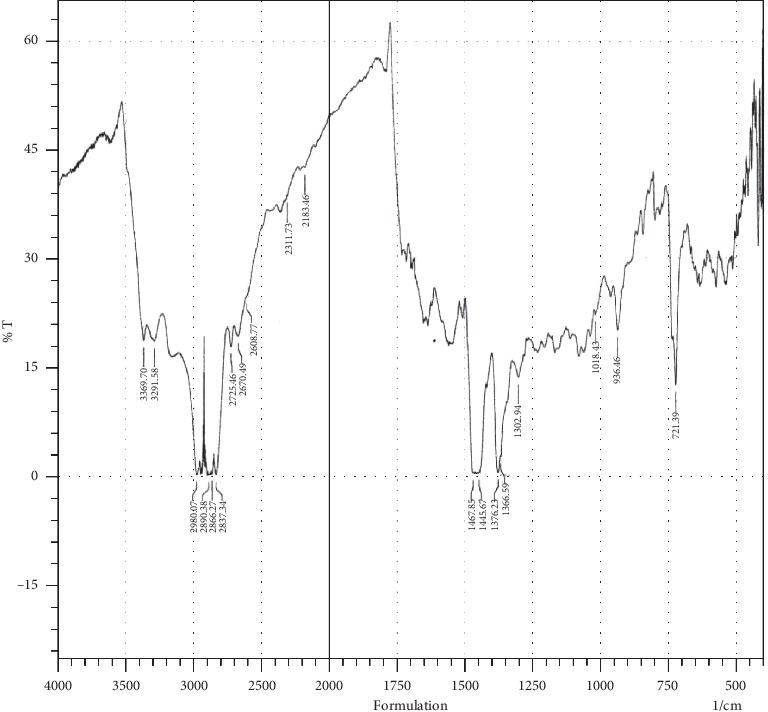
FT-IR spectra of the mixture of MET HCl and GLB with excipients.

The FT-IR spectra of the physical mixture of MET HCl, GLB, PEG 6000, and SLS are shown in Figure 2. The characteristic peaks of MET HCl are at 3369.70 cm^−1^ and 3291.58 cm^−1^ (N-H stretching), 2890.38 cm^−1^ and 2837.34 cm^−1^ (C-H stretching), 1376.23 cm^−1^ (N-H bending), and 1018.43 cm^−1^ (C-N stretching). In case of GLB, C-H stretching at 2980.07 cm^−1^ and 2866.27 cm^−1^, O=S=O stretching at 2311.73 cm^−1^, N-H deformation at 1467.85 cm^−1^ and 1445.67 cm^−1^, and C-N stretching at 1366.59 cm^−1^ are observed. The presence of all typical peaks of MET HCl and GLB in the mixture and the absence of the major shift in the FT-IR spectra indicate that there is no incompatibility between the drugs and the excipients used in the formulations.

### 4.2. Preliminary Studies

Preliminary studies were conducted to select the most critical factors that affect the dependent variables [14]. A previous study found that concentration of the binder and superdisintegrant from the formulation variables and compression force from the processing variables are the most important factors that affect the different parameters of orodispersible tablets (i.e., disintegration time, friability, hardness, and drug release) [15]. In this study, it was found that the PEG 6000 concentration, crospovidone concentration, and compression force significantly affected disintegration time and friability.

### 4.3. Granule and Tablet Characteristics of the Preliminary Formulations

The bulk density was between 0.41 ± 0.00 and 0.59 ± 0.02 g/mL and tapped density between 0.47 ± 0.00 and 0.63 ± 0.00 g/mL. All formulations had Hausner's ratio less than 1.18, Carr's index between 6.30% ± 0.60% and 15.78% ± 0.20%, and angle of repose in the range of 25.00 ± 1.10° to 27.00 ± 2.30°. According to these results, granules from all batches had good to excellent flow properties [14].

The thickness of the tablets ranged between 3.47 ± 0.05 and 3.89 ± 0.14 mm and diameter between 9.95 ± 0.00 mm and 10.03 ± 0.00 mm. The hardness of the tablets was between 4.11 ± 0.24 kg/cm^2^ and 10.15 ± 0.41 kg/cm^2^. These tablets have a short wetting time between 28 and 96 seconds. As wetting time has a direct relation with disintegration time, this short wetting time in the present study is a good indicator for fast disintegration of the preliminary ODTs [16]. As shown in Table 5, the weight variation and drug content of all preliminary batch tablets were within the acceptable pharmacopoeial range. According to the USP, MET HCl tablets should contain not less than 95% and not more than 105% of the stated amount and GLB tablets should contain not less than 90% and not more than 110% [12]. Thus, all preliminary formulated tablets passed the drug content determination specification.

### 4.4. Effects of Sodium Lauryl Sulphate

**Figure 3:**
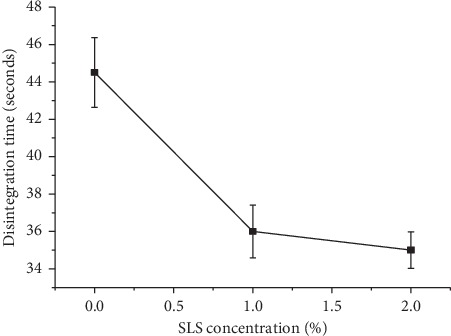
Effects of SLS on the disintegration time of the preliminary FDC ODTs.

Sodium lauryl sulphate (SLS), a surface active agent, was added to enhance the wettability of preliminary tablets which in turn facilitates the disintegration of ODTs [17]. In order to investigate the effect of SLS on the disintegration time, preliminary tablets were prepared in the absence of SLS and with 1% SLS and 2% SLS, as shown in Figure 3, before the preliminary study. The disintegration time of these tablets decreased significantly (*P* < 0.05) when SLS concentration increased from 0 to 1%. However, further increasing the concentration to 2% resulted in an insignificant increase in the disintegration time. A similar phenomenon was reported by Rakesh et al. [18] where the addition of SLS significantly decreased the disintegration time up to the concentration of 0.8%; nevertheless, further increment led to an insignificant decrease of disintegration time. Therefore, SLS concentration of 1% was used for further study.

### 4.5. Effects of Polyethylene Glycol 6000

Polyethylene glycol 6000 is a meltable hydrophilic polymer which acts as a binder to obtain tablets of sufficient mechanical strength. Moreover, it enhances the dissolution characteristics of poorly water-soluble compounds by the melt granulation technique [19]. Increasing the PEG 6000 concentration from 2 to 7% significantly increased the disintegration time from 13 to 83 seconds (*P*=0.0026), as shown in Table 6. This may be attributed to the strong binding action of PEG 6000 [20]. Furthermore, at a higher PEG 6000 concentration, a thick gel layer will be formed which acts as a barrier to the penetration of the disintegration medium leading to a longer disintegration time [21].

Similarly, there was a significant decrease in tablet friability, 0.23 to 1.12% (*P*=0.0158), with the increased PEG 6000 concentration. This is probably the result of better plasticization of the compressed granules by melted PEG 6000 which allows for more effective spreading of the binder around particles [21].

Although the tablets contain glibenclamide which is a poorly water-soluble drug, the percentage of drug release of all tablet formulations was between 89.25% and 95.3% for MET HCl and 84.3% and 91.18% for GLB after 30 minutes of dissolution time. These enhanced releases of tablets even for a poorly water-soluble drug (GLB) could be due to the effect of solid dispersions of drugs on a hydrophilic carrier (PEG 6000) matrix which might have enhanced the dissolution rate [19].

### 4.6. Effects of Crospovidone

As shown previously in Table 6, the disintegration time significantly decreased when the concentration of crospovidone increased from 2% to 10% (*P*=0.0005). This effect could be associated with the porous particle morphology of crospovidone which facilitates the intake of water into the tablet, resulting in swelling, rupture of interparticle bonds, and final disintegration of the tablets [22]. In the current study, crospovidone concentration did not result in a significant change (*P* > 0.05) in both friability and dissolution values. These results are in agreement with the reports by Salem and Badwan [23].

### 4.7. Effects of Compression Force

In order to investigate the effect of compression force, preliminary tablets were formulated at compression forces of 5 kN and 15 kN. As depicted above in Table 6, the friability of tablets significantly decreased as the compression force increased (*P*=0.0134). This might be due to the plastic deformation ability of microcrystalline cellulose and PEG 6000 at high compression force which could facilitate the formation of harder and less friable tablets as rationalized by Ilic et al. [24]. In contrast, the disintegration time significantly increased (*P*=0.0045) with compression force. This effect could be attributed to increasing the tablet strength and density which hinder the disintegration medium to penetrate the tablet structure [25].

### 4.8. In Vitro Drug Release

**Figure 4:**
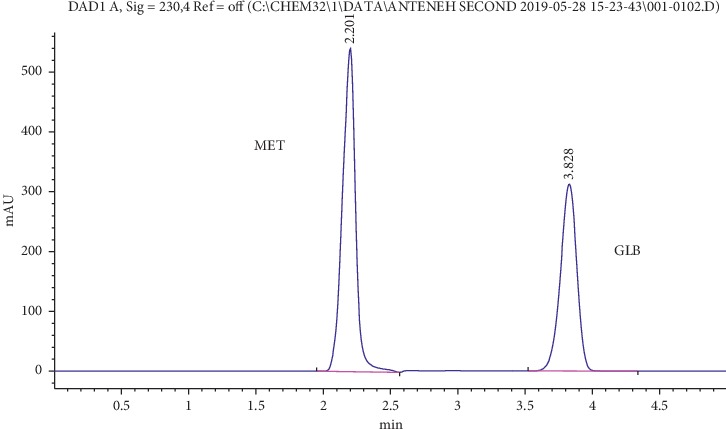
Typical chromatogram of a mixture of MET HCl (150 *μ*g/mL) and GLB (150 *μ*g/mL) at retention times of 2.201 and 3.828 minutes, respectively.

The percentage of drug release was calculated based on the standard chromatogram depicted in Figure 4. The dissolution study of all tablets prepared in the preliminary study indicated that more amount of drug was released within 30 minutes compared to the minimum value (80%) of pharmacopoeial specifications for both drugs, as presented in Table 6. These increased drug release values of tablets even in the presence of a poorly water-soluble drug (GLB) might be due to the effect of solid dispersions of drugs on a highly hydrophilic carrier (PEG 6000) matrix. It has been shown that solid dispersion is one of the methods to enhance the solubility and dissolution rate of poorly water-soluble drugs [19]. Moreover, the micronized form of glibenclamide used in this study might have contributed to the increased dissolution rate as decreased particle size increases the surface area, which in turn increases the dissolution rate [26].

Although the drug release values were increased compared to the pharmacopoeial specifications, there were no significant changes (*P* > 0.05) in the percentage of drug release of both drugs as PEG 6000, crospovidone, and compression force changed from the minimum to the maximum level. These findings were slightly different from previously observed findings in other studies which showed that as the PEG 6000 concentration increased, drug release also increased for immediate release tablets [27]. This limited effect of PEG 6000 on the drug release might be due to the fast disintegration nature of the tablets and micronized form of glibenclamide used in this study [28].

### 4.9. Optimization Study

From the preliminary study, it was found that the concentration of PEG 6000, concentration of crospovidone, and level of compression force significantly affected the disintegration time and friability of tablets. Therefore, the effects of these independent variables on the disintegration time and friability of tablets were further studied with the central composite design (CCD). The compositions of independent variables in the 19 formulations of fixed-dose combinations of metformin HCl and glibenclamide ODT as given by the CCD are shown in Table 7.

The disintegration time and friability of 19 formulations prepared as per experimental conditions of the CCD are shown in Table 8. These results were input into the Design-Expert software for the optimization analysis.

### 4.10. Response Model Selection

A mathematical model was selected to find suitable values of the controllable factors in the formulation of a monolithic FDC of MET HCl and GLB ODTs. The selection was based on the comparisons of several statistical parameters including the coefficient of variation (CV), multiple correlation coefficient (*R*^2^), adjusted multiple correlation coefficient (adjusted *R*^2^), and predicted residual sum of squares (PRESS), provided by Design-Expert® software [28]. Accordingly, the quadratic model and linear model were selected for friability and disintegration time, respectively.

The goodness of fit of the model was validated by the determination coefficient (*R*^2^). The *R*^2^ value of the quadratic model is 0.9831. This value indicates that 98.3% of the total variation in tablet friability is attributed to the independent variables and only 1.7% of the total variation cannot be explained by the model [29]. The adjusted *R*^2^ value of 0.966 was in a reasonable distance from the determination coefficient (within 0.20 of each other) [30]. This model also had a low PRESS value (0.22) compared to other models (linear and 2FI). In case of the linear model, the *R*^2^ value of 0.8221 indicates that 82.2% of variation of disintegration time is explained by the model. The predicted *R*^2^ value of 0.7100 is in reasonable agreement with the adjusted *R*^2^ value of 0.7865.

Model adequacy checking is also important to check the relationship between dependent and independent variables, statistical independence of errors, constant variance of errors, and normality of the error distribution based on the ANOVA result [31, 32]. As shown in Table 9, both models are statistically significant. Furthermore, the ANOVA showed that the main effects, PEG 6000, crospovidone, and compression force, were significant model terms for the linear model of disintegration time. For the quadratic model, the concentration of PEG 6000, compression force, the quadratic effect of PEG 6000 and compression force, and the interaction effect of PEG 6000 and compression force were significant model terms. Insignificant model terms were reduced with the backward elimination procedure to improve the model prediction efficiency. The lack-of-fit test was insignificant for both models which indicates models are sufficient to explain the observed data. The adequate precision (signal-to-noise ratio) values of 29.836 for friability and 16.035 for disintegration time obtained were very high compared to the desired value greater than 4 [30]. The residuals of both models also well behaved and were normally distributed. Based on this evidence, it is reasonable to conclude that both models were fairly accurate and could be used for further analysis.

Therefore, mathematical regression models (equations (11) and (12)) were generated for both responses in terms of coded factors and using model term coefficients:

(10) $$\begin{array}{c} \text{friability}=0.273-0.187*A-0.207*C+0.075*AC+0.0458A^{2}+0.228C^{2}, \end{array}$$

(11) $$\begin{array}{c} \text{disintegration time}=46.9+15*A-26.8*B+12.1*C, \end{array}$$

where *A* is PEG 6000, *B* is crospovidone, and *C* is compression force.

**Figure 5:**
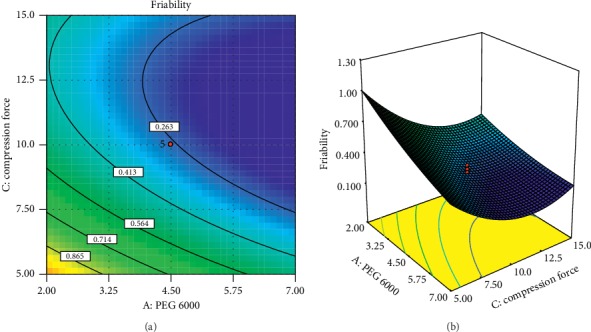
Contour plot (a) and response surface plot (b) of PEG 6000 and level of compression force on friability.

**Figure 6:**
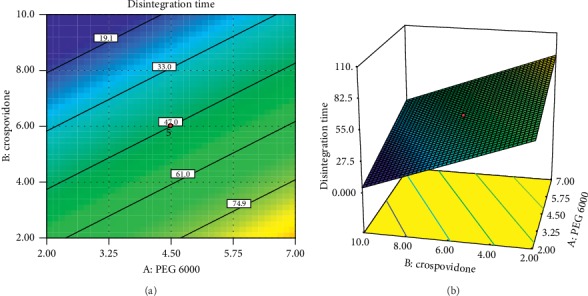
Contour plot (a) and response surface plot (b) of PEG 6000 and crospovidone on disintegration time.

A positive sign before a factor in polynomial equations represents that the response increases with the factor. On the contrary, a negative sign means the response and factors have a reciprocal relation. From equations (10) and (11), it can be observed that PEG 6000 has a negative effect on the friability of tablets and a positive effect on the disintegration time of tablets. This indicates that increasing the PEG 6000 concentration decreases tablet friability and increases the disintegration time of tablets. Similarly, increasing the level of compression force decreases friability and increases disintegration time. On the contrary, the interaction of PEG 6000 and compression force has a positive impact on the friability of tablets. The crospovidone concentration has a negative effect on disintegration time which indicates increasing the concentration of crospovidone decreases the disintegration time. The coefficient before the factor implies the change in response when each factor is changed by one unit while keeping other factors constant [22]. Moreover, the value of the coefficient indicates the extent of the factor effect; the higher the value, the greater the effect of the factor on the response variable. Accordingly, for disintegration time, the concentration of crospovidone (−26.8) has a greater effect than PEG 6000 and compression force, whereas for the friability, the compression force (−0.207) has a greater effect than PEG 6000. These phenomena can be clearly seen in 2D contour and 3D response surface plots in Figures 5 and 6.

### 4.11. Simultaneous Optimization of Friability and Disintegration Time

After producing polynomial equations for each model to relate the independent and response variables, the formulation was optimized for the two responses simultaneously. Both numerical and graphical optimization techniques of Design-Expert software were used to obtain the optimum condition. The main goal of the optimization process was to get lower friability and disintegration time of tablets within the pharmacopoeial range. The goal, lower and upper limit criteria, and importance of all factors and response during optimizations are shown in Table 10.

**Figure 7:**
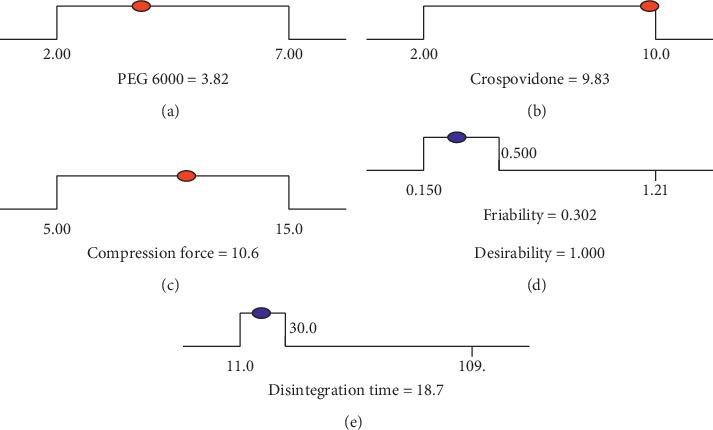
Desirability ramp for numerical optimization of five goals, namely, (a) PEG 6000, (b) crospovidone, (c) compression force, (d) friability, and (e) disintegration time.

Numerical optimization searches the design space, using the developed regression model to find the factor settings that optimize any combination of one or more goals. It finds a point that maximizes this desirability function [33]. To find the global (overall) desirability function, the Design-Expert software carries out thousands of calculations, and finally, it provides the maximum desirability score and the sets of conditions on which it has arrived. Consequently, the predicted optimum values and the corresponding levels of parameters according to the set goals were obtained, as depicted in Figure 7. A dot indicates the best solution found by the Design-Expert software.

The desirability value ranges between 0 and 1: zero value indicates undesirable quality and 1 points out that the quality of the associated response is optimal [32]. In this study, the overall desirability function of both responses was obtained from the individual desirability functions and found to be 1.00 as calculated from the optimal point obtained (friability = 0.302 and disintegration time = 18.7) based on the following equation:

(12) $$\begin{array}{c} D={\left(d1^{p1}\;d2^{p2}d3^{p3}\ldots di^{pi}\right)}^{\left(1/\sum pi\right)}, \end{array}$$

where *i* is the number of responses, *di* is the individual desirability functions, and *pi* is the relative importance of the *i*^th^ response as compared to others. Importance (*pi*) varies from 1 to 5, from least to most important, respectively.

**Figure 8:**
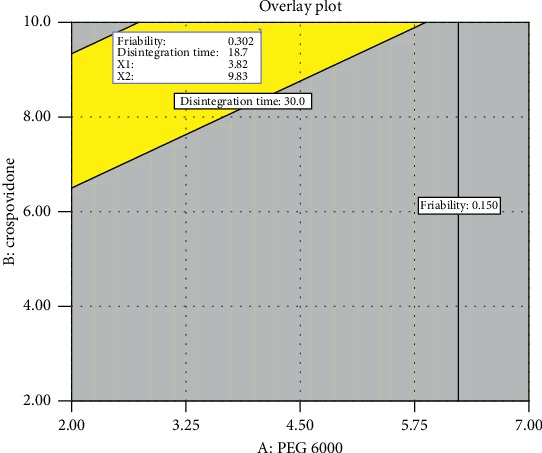
Optimum region identified by overlaying plots of friability and disintegration time as functions of PEG 6000 and crospovidone at 10.6 kN compression force.

Graphical optimization is also possible to obtain a solution by drawing overlay plots of constraint functions and the objective function. It allows for a visual selection of the optimum conditions according to setting criteria [34].  Figure 8 shows the overlay plot in which the yellow area represents the area complying with the imposed criteria. The area identified by yellow colour was preferred to be a representative of the optimized area corresponding to 3.82% of PEG 6000 and 9.83% of crospovidone. With these conditions, the software predicts a friability value of 0.302% and disintegration time of 18.7 seconds.

### 4.12. Confirmation Test

The confirmation test was done to check the validity of optimized formulation obtained from the predicted value. The test was conducted in triplicate using the predicted optimal values of the independent variables (PEG 6000 = 3.82%, crospovidone = 9.83%, and compression force = 10.6 kN). The prepared tablets were evaluated for friability and disintegration time. As shown in Table 11, the percentage error obtained from optimization results was less than 5% for both responses; this indicates that the predicted values were in agreement with experimental results [35].

The optimized formulation was characterized for its granule and tablet properties. As presented in Table 12, the formulation has good to excellent flow property of granules and tablets with good quality according to the pharmacopoeial specification.

**Figure 9:**
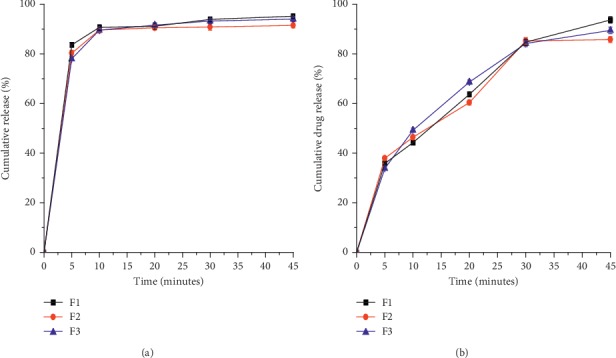
Release profiles of metformin HCl (a) and GLB (b) from three batches of the optimized monolithic fixed-dose combination orodispersible tablets.

The drug release profile of the optimized formulations was also investigated, as shown in Figure 9. All of the three ODT formulations released more than 80% of the drug before 30 minutes. This is in agreement with the USP specification which states that not less than 80% of MET HCl and GLB should be released within 30 minutes from tablets [12]. The ANOVA results of the release profiles based on dissolution efficiency values of the three batches, 91.13 ± 0.26, 90.84 ± 0.41, and 93.23 ± 0.82 for MET HCL and 89.48 ± 0.91, 85.25 ± 0.33, and 87.73 ± 0.57 for GLB, revealed that there was no statistically significant difference (*P* > 0.05) in release profiles of the formulations.

## 5. Conclusion

The FT-IR studies indicated that there are no incompatibility problems between MET HCl and GLB as well as between the excipients used in the proposed formulation. The preliminary studies showed that both formulation variables (PEG 6000 and crospovidone) and process variable (level of compression force) have a significant influence on the characteristics of the prepared tablets. The PEG 6000 concentration and level of compression force were the determinant factors for tablet friability, whereas all the three factors (PEG 6000 concentration, crospovidone concentration, and compression force) were the determinant factors for tablet disintegration time.

The CCD from the RSM was employed to optimize PEG 6000, crospovidone, and compression force with respect to friability and rapid disintegration time. Accordingly, the desired optimum condition was obtained at 3.82% of PEG 6000, 9.83% of crospovidone, and 10.6 kN compression force. With these optimum conditions, the friability and disintegration time were predicted to be 0.302% and 18.7 seconds, respectively. The experimental values of the monolithic FDC of MET HCl and GLB ODTs prepared under the optimum conditions were within 5% of the predicted values. The optimized tablets fulfilled all pharmacopoeial specifications.

According to the results of this study, a monolithic FDC of MET HCl and GLB ODTs with adequate mechanical strength, faster disintegration time, and enhanced dissolution rate can be successfully formulated. Therefore, the formulated FDC ODT can be used as a potential option to solve pill burden and swallowing problem for type II DM patients.

## Figures and Tables

**Table 1:** Composition of the preliminary fixed-dose combination of orodispersible tablets.

Code	Ingredients (mg)									Compression force (kN)
	MET	GLB	PEG 6000	CPV	Aspartame	SLS	CSD	Mg.st	MCC	
Fp1	250	2.5	7	7	1.75	3.5	1	3.5	73.75	5
Fp2	250	2.5	24.5	7	1.75	3.5	1	3.5	56.25	5
Fp3	250	2.5	7	35	1.75	3.5	1	3.5	45.75	5
Fp4	250	2.5	24.5	35	1.75	3.5	1	3.5	28.25	5
Fp5	250	2.5	7	7	1.75	3.5	1	3.5	73.75	15
Fp6	250	2.5	24.5	7	1.75	3.5	1	3.5	56.25	15
Fp7	250	2.5	7	35	1.75	3.5	1	3.5	45.75	15
Fp8	250	2.5	24.5	35	1.75	3.5	1	3.5	28.25	15

Fp: preliminary formulation; CPV: crospovidone; CSD: colloidal silicon dioxide; Mg.st: magnesium stearate; MCC: microcrystalline cellulose.

**Table 2:** System suitability parameters.

Parameter	MET	GLB	Acceptable limits
Retention time (RT)	2.206 ± 0.046	3.828 ± 0.058	
	RSD = 0.002	RSD = 0.003	RSD<1%
Theoretical plates (*N*)	2,300	4,356	*N* > 2000
Tailing factor (*T*)	1.65 + 0.02	1.134 + 0.04	*T* < 2

**Table 3:** Experimental levels of the independent variables for preliminary formulations.

Variables	Levels	
	Minimum (−1)	Maximum (+1)
PEG 6000 (%)	2	7
Crospovidone (%)	2	10
Compression force (kN)	5	15

**Table 4:** Experimental levels of the independent variables for optimizing the fixed-dose combination of metformin HCl and glibenclamide orodispersible tablets.

Variables	Levels				
	−*α*	−1	0	+1	+*α*
PEG 6000 (%)	0.296	2	4.5	7	8.70
Crospovidone (%)	−0.727	2	6	10	12.7
Compression force (kN)	1.59	5	10	15	18.40

*α* = 1.68179.

**Table 5:** Tablet properties of the preliminary FDC of metformin HCl and glibenclamide ODTs.

Formulation code	Hardness (kg/cm^2^)	Weight (mg)	Wetting time (seconds)	Drug content (%)	
				MET	GLB
Fp1	5.30 ± 0.32	349.70 ± 1.88	57 ± 0.81	98.78 ± 1.33	100.83 ± 0.46
Fp2	8.05 ± 0.13	347.60 ± 2.59	80 ± 1.76	100.64 ± 1.08	102.29 ± 0.38
Fp3	4.11 ± 0.24	350.20 ± 2.25	28 ± 0.87	100.09 ± 1.11	104.10 ± 0.81
Fp4	7.81 ± 0.92	349.90 ± 2.28	49 ± 0.44	98.23 ± 0.88	101.84 + 0.22
Fp5	7.72 ± 0.06	349.6 ± 1.64	87 ± 2.05	97.51 ± 1.12	103.36 ± 0.43
Fp6	10.15 ± 0.41	349.50 ± 1.78	96 ± 0.42	100.26 ± 0.69	100.12 ± 0.61
Fp7	8.42 ± 0.28	352.90 ± 1.37	39 ± 0.49	97.80 ± 0.83	99.36 ± 0.26
Fp8	9.34 ± 0.22	349.00 ± 1.63	65 ± 0.79	98.27 ± 1.41	101.91 ± 0.36

**Table 6:** Effects of independent variables on friability, disintegration time, and percentage of drug release in the first 30 minutes (mean ± SD).

Formulation code	Friability (%)	Disintegration time (seconds)	DR (%) in 30 min	
			MET	GLB
Fp1	1.05 ± 0.26	45 ± 0.73	91.87 ± 1.31	89.72 ± 1.81
Fp2	0.73 ± 0.03	68 ± 1.07	92.05 ± 1.02	90.85 ± 1.32
Fp3	1.12 ± 0.35	13 ± 0.78	89.40 ± 2.14	91.18 ± 0.95
Fp4	0.80 ± 0.02	37 ± 1.78	95.33 ± 0.98	85.30 ± 1.14
Fp5	0.50 ± 0.05	76 ± 1.07	89.25 ± 1.23	86.96 ± 2.10
Fp6	0.23 ± 0.01	83 ± 0.89	94.26 ± 1.64	84.30 ± 1.81
Fp7	0.99 ± 0.26	28 ± 0.63	91.59 ± 0.67	89.36 ± 0.86
Fp8	0.30 ± 0.10	45 ± 0.73	93.32 ± 1.59	86.31 ± 1.91

DR (%) = percentage of drug release.

**Table 7:** Composition of 19 formulations based on the central composite design.

Formulation code	Point type	Independent variables		
		PEG 6000 (%)	Crospovidone (%)	Compression force (kN)
Fd1	Axial	4.5(0)	6(0)	18.41(+*α*)
Fd2	Center	4.5(0)	6(0)	10(0)
Fd3	Factorial	2(−1)	2(−1)	5(−1)
Fd4	Center	4.5(0)	6(0)	10(0)
Fd5	Factorial	7(+1)	2(−1)	5(−1)
Fd6	Factorial	2(−1)	10(+1)	15(+1)
Fd7	Axial	4.5(0)	6(0)	1.59 *α*
Fd8	Factorial	2(−1)	2(−1)	15(+1)
Fd9	Factorial	2(−1)	10(+1)	5(−1)
Fd10	Factorial	7(+1)	2(−1)	15(+1)
Fd11	Axial	4.5(0)	12.7(+*α*)	10(0)
Fd12	Center	4.5(0)	6(0)	10(0)
Fd13	Factorial	7(+1)	10(+1)	15(+1)
Fd14	Axial	8.7 (+*α*)	6(0)	10(0)
Fd15	Axial	4.7(0)	2(−*α*)	10(0)
Fd16	Axial	0.296(−*α*)	6(0)	10(0)
Fd17	Center	4.5(0)	6(0)	10(0)
Fd18	Center	4.5(0)	6(0)	10(0)
Fd19	Factorial	7(+1)	10(+1)	5(−1)

Fd = formulation design based on the CCD.

**Table 8:** Experimental design batches for FDC ODTs with response parameters (mean ± SD).

Formulation code	Responses	
	Friability (%)	Disintegration time (seconds)
Fd1	0.65 ± 0.02	64 ± 1.78
Fd2	0.28 ± 0.01	26 ± 0.89
Fd3	0.98 ± 0.03	53 ± 2.68
Fd4	0.26 ± 0.02	34 ± 1.78
Fd5	0.47 ± 0.00	73 ± 1.57
Fd6	0.51 ± 0.01	19 ± 0.80
Fd7	1.21 ± 0.13	11 ± 0.89
Fd8	0.36 ± 0.01	84 ± 2.68
Fd9	1.13 ± 0.11	13 ± 0.88
Fd10	0.18 ± 0.00	93 ± 3.57
Fd11	0.23 ± 0.00	14 ± 0.89
Fd12	0.30 ± 0.00	35 ± 0.78
Fd13	0.16 ± 0.00	42 ± 1.78
Fd14	0.15 ± 0.00	102 ± 2.68
Fd15	0.20 ± 0.00	109 ± 4.47
Fd16	0.68 ± 0.03	16 ± 0.82
Fd17	0.35 ± 0.01	49 ± 0.68
Fd18	0.31 ± 0.00	33 ± 1.78
Fd19	0.51 ± 0.01	23 ± 0.89

Fd = formulation design based on the CCD.

**Table 9:** Summary of ANOVA results of the response surface quadratic model for friability and the linear model for disintegration time.

Responses	Source	Sum of squares	df	Mean square	*F* value	*P* value	Remark
Friability	Model	1.83	9	0.367	74.4	<0.0001	Significant
	A-PEG 6000	0.477	1	0.477	96.8	<0.0001	Significant
	C-compression force	0.583	1	0.583	118	<0.0001	Significant
	AC	0.0450	1	0.0450	9.14	0.00980	Significant
	A^2^	0.0293	1	0.0293	5.95	0.0298	Significant
	C^2^	0.725	1	0.725	147	<0.0001	Significant
	Residual	0.0640	13	0.00493			Significant
	Lack of fit	0.0594	9	*0.00660*	5.74	0.0539	Insignificant
	Pure error	*0.00460*	4	*0.00115*			
	Core total	1.90	18				
Disintegration time	Model	14868.94	3	4956.31	23.10	<0.0001	Significant
	A-PEG 6000	3075.78	1	3075.78	14.34	0.0018	Significant
	B-crospovidone	9796.39	1	9796.39	45.66	<0.0001	Significant
	C-compression force	1996.77	1	1996.77	9.31	0.0081	Significant
	Residual	3218.00	15	214.53			
	Lack of fit	2936.80	11	266.98	3.80	0.1046	Insignificant
	Pure error	281.20	4	70.30			
	Core total	18086.95	18				

**Table 10:** Constrains of factors and responses for optimization of the FDC of MET HCl and GLB ODTs.

Name	Goal	Lower limit	Upper limit	Lower weight	Upper weight	Importance
PEG 6000 (%)	Range	2	7	1	1	3
Crospovidone (%)	Range	2	10	1	1	3
Compression force (kN)	Range	5	15	1	1	3
Friability (%)	Range	0.15	0.5	1	1	3
Disintegration time (sec)	Range	11	30	1	1	3

**Table 11:** Predicted and experimental response values and percentage error obtained at optimal levels of the factors.

Responses	Predicted value	Experimental value	% error
Friability (%)	0.302	0.294 ± 0.010	2.72
Disintegration time (sec)	18.7	19.20 ± 0.84	3.64

Evaluation of the optimized FDC of MET HCl and GLB ODTs.

**Table 12:** Granule and tablet properties of the optimized FDC of MET HCl and GLB formulation.

Parameters					Experimental value	
*Granule properties*						
Bulk density (gm/mL)					0.53 + 0.02	
Tapped density (gm/mL)					0.61 + 0.00	
Hausner's ratio					1.15 + 0.00	
Carr's index (%)					13.11 + 0.90	
Angle of repose (°)					26.00 + 0.80	
*Tablet properties*						
Formulation code	Diameter (mm)	Thickness (mm)	Hardness (kg/cm^2^)	Weight (mg)	Assay (%)	
					MET	GLB
F1	10.02 ± 0.00	3.83 ± 0.04	7.83 + 0.12	349.30 ± 1.6	98.08 + 0.92	104.34 + 0.21
F2	10.04 ± 0.02	3.84 ± 0.02	7.82 + 0.68	348.90 ± 2.0	101.05 + 0.61	101.59 + 0.13
F3	9.92 ± 0.14	3.83 ± 0.04	7.84 + 0.05	348.70 ± 2.4	96.79 + 0.32	100.68 + 0.74

## Data Availability

The data used to support the findings of this study are included within the article.
